# Exploring the mechanism of immediate analgesic effect of 1-time tuina intervention in minor chronic constriction injury rats using RNA-seq

**DOI:** 10.3389/fnins.2022.1007432

**Published:** 2022-10-04

**Authors:** Hourong Wang, Zhifeng Liu, Tianyuan Yu, Yingqi Zhang, Yajing Xu, Yi Jiao, Qian Guan, Di Liu

**Affiliations:** ^1^School of Acupuncture-Moxibustion and Tuina, Beijing University of Chinese Medicine, Beijing, China; ^2^Department of Tuina and Pain Management, Dongzhimen Hospital of Beijing University of Chinese Medicine, Beijing, China; ^3^Department of Acupuncture, Oriental Hospital of Beijing University of Chinese Medicine, Beijing, China

**Keywords:** neuropathic pain, tuina, RNA sequencing, dorsal root ganglia, spinal dorsal horn, analgesia

## Abstract

Previous studies have proved and investigated the mechanism of the analgesic effect of tuina treatment on neuropathic pain. The purpose of this study was to analyze changes in gene expression in the dorsal root ganglia (DRG) and spinal dorsal horn (SDH) after 1-time tuina intervention to investigate the immediate analgesic mechanism by tuina. An improvement in nociceptive behavior in minor chronic constriction injury (CCI) rats after 1-time tuina was observed. 1-time tuina was more effective in the amelioration of thermal hyperalgesia, but no changes were found in the ultrastructure of DRG and SDH. Sixty-five differentially expressed genes (DEGs) modulated by tuina were detected in the DRG and 123 DEGs were detected in the SDH. Potential immediate analgesic mechanisms of tuina were analyzed by the Kyoto Encyclopedia of Genes and Genomes. DEGs were enriched in 75 pathways in DRG, and 107 pathways in SDH. The immediate analgesic mechanism of tuina is related to the calcium signaling pathway, thermogenesis, and regulation of lipolysis in adipocytes.

## Introduction

Neuropathic pain (NP) is a syndrome caused by injury or disease of the somatosensory nervous system, either in the periphery or central, and is characterized by spontaneous pain, hyperalgesia, allodynia, and paresthesia (Baron et al., 2010). NP has a significant influence on the physical and psychological health of patients, and the prevalence in the whole population is approximately 10%, which varies by disease. Due to the complicated pathogenesis, current treatments such as medication did not show obvious effects (Schaefer et al., 2014; Fayaz et al., 2016; Scholz et al., 2019; Zheng et al., 2020).

Tuina is an alternative medical therapy which is safe and has virtually no side effects (Field, 2016). Clinical studies have proved that tuina has either immediate or cumulative analgesic effects, which can ameliorate the symptoms of NP (Gok Metin et al., 2017; Izgu et al., 2019). Its cumulative analgesic mechanism is based on the down-regulation of inflammatory cytokines (such as tumor necrosis factor-α, interleukin-1, and interleukin-1β) and the inhibition of microglial activation (Liu et al., 2021). Our previous studies confirmed the effectiveness of tuina analgesia, and found differentially expressed genes (DEGs) in DRG and SDH of rats with sciatic nerve injury after 20-time tuina treatment, which were mainly related to regulation of protein binding, response to pressure, and neuron projection (Lv, 2020; Lv et al., 2020a). However, the immediate analgesic mechanism of tuina is unknown. Clinically, 1 or 2 time tuina treatment can effectively relieve pain, achieve immediate analgesia and prevent disease progression in patients (Li et al., 2004; Qing et al., 2022). Therefore, searching for immediate effective targets and exploring mechanisms of tuina analgesia can provide new evidence and methods for the therapeutic targets of NP, as well as help tuina to be accepted and applied in more countries and regions.

As the sensory neurons of the first and second levels, the dorsal root ganglia (DRG) and the spinal dorsal horn (SDH) are the primary portals for nerve afferents and integration of pain information. They are important for both inducing central sensitization and processing NP development (Simeoli et al., 2017; Inoue and Tsuda, 2018; Shepherd et al., 2018). Here, we investigate the immediate analgesic mechanism of 1-time tuina and changes in RNA expression in DRG and SDH using the minor chronic constriction injury (CCI) model, which is a recognized model for simulating clinical peripheral neuropathic pain (Jaggi et al., 2011). To this end, we behaviorally assessed mechanical and thermal allodynia changes after the tuina treatment and non-treatment conditions in the CCI rat model. We also analyzed the gene expression differences and functions before and after intervention in the DRG and SDH of the surgical side by RNA-Seq.

## Results

### Changes in symptoms of hyperalgesia and allodynia in minor chronic constriction injury rats

**FIGURE 1:**
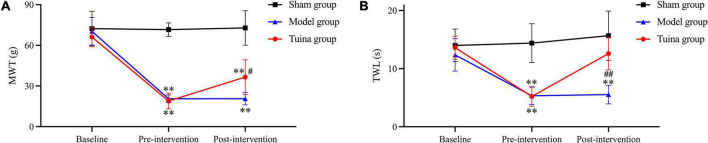
Results of behavioral tests of rats in each group. **(A)** MWT; **(B)** TWL. Sham group (*n* = 6); model group (*n* = 6); tuina group (*n* = 6). ^**^P < 0.01, *vs.* the sham group; ^#^*P* < 0.05, ^##^*P* < 0.01, *vs.* the model group.

The rats were in good condition and recovered well from the surgical incision. The posture of rats in the sham group was normal when walking and resting. Rats in the model and tuina group had curled hind paws on the surgical side, limping when walking, and mostly lying on the left side when resting. There was no statistical difference in behavior tests between groups before modeling, and before intervention. Mechanical withdrawal threshold (MWT) and thermal withdrawal latency (TWL) were decreased in both the model (*P* = 0.00, *P* = 0.00) and tuina groups (*P* = 0.00, *P* = 0.00) compared with the sham group. After a 1-time tuina intervention, MWT and TWL increased in the tuina group (*P* = 0.014, *P* = 0.00) compared with the model group (Figure 1 and Table 1).

**TABLE 1:** Results of behavioral tests of rats in each group.

	MWT			TWL		
		
	Baseline	Pre-intervention	Post-intervention	Baseline	Pre-intervention	Post-intervention
Sham group	72.36 ± 12.7	71.61 ± 4.95	72.83 ± 12.7	14 ± 2.81	14.39 ± 3.33	15.68 ± 4.24
Model group	70.33 ± 10.17	20.56 ± 2.84	20.64 ± 4.56	12.37 ± 2.79	5.33 ± 1.49	5.53 ± 1.6
Tuina group	66.18 ± 7.19	18.91 ± 5.78	36.53 ± 12.78	13.61 ± 1.98	5.22 ± 1.68	12.58 ± 2.78

### Changes in the ultrastructure of dorsal root ganglia and spinal dorsal horn

**FIGURE 2:**
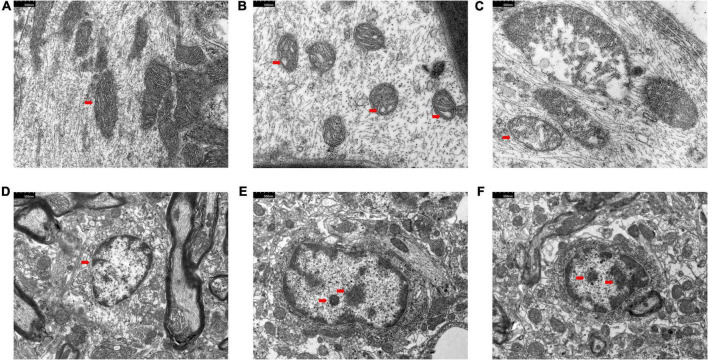
Electron microscopy results of DRG and SDH. Sham group (*n* = 3); model group (*n* = 3); tuina group (*n* = 3). Panels **(A–C)** were DRG; the arrow showed the mitochondrial vacuoles; panels **(D–F)** were SDH; the arrow shows the chromatin aggregation. **(A,D)** Sham group; **(B,E)** Model group; **(C,F)** Tuina group.

The electron microscopy results of DRG in the sham group showed that the mitochondrial morphology was intact, and the mitochondrial matrix was homogeneous (Figure 2A). The results of the model and tuina groups showed mitochondrial swelling, disorganized mitochondrial cristae arrangement with matrix dissolution and dilated mitochondria with occasional vacuolization (Figures 2B,C). The electron microscopic results of SDH in the sham group showed the nuclear membrane was smooth and intact, the perinuclear gap was not significantly widened, and the chromatin was sparse (Figure 2D). The images of the model and tuina groups showed that the nucleus was irregular in shape, the double-layered nuclear membrane was intact, the perinuclear gap was not significantly widened, and the chromatin was aggregated (Figures 2E,F).

### Bioinformatic analysis of dorsal root ganglia and spinal dorsal horn on the surgical side of rats

#### Results of the sample quality control

The results of performing quality control on the raw data indicated that the data were qualified and ready for subsequent analysis. We obtained a total of 920 million reads, including an average of 49.84 million reads per DRG sample and 53.32 million reads per SDH sample. The results of the error rate, Q20 and Q30 met the quality control standards, which showed that the sequencing results were reliable and credible (Table 2).

**TABLE 2:** Sample sequencing information and quality control results.

Sample	Raw reads	Clean reads	Error rate (%)	Q20 (%)	Q30 (%)
DRG_Sham_1	48158012	47760586	0.0243	98.31	94.9
DRG_Sham_2	56223240	55863814	0.0237	98.52	95.44
DRG_Sham_3	46544026	46183596	0.0241	98.36	95.02
DRG_Model_1	47859852	47517974	0.0236	98.56	95.58
DRG_Model_2	45210228	44920418	0.0238	98.51	95.42
DRG_Model_3	54200106	53876928	0.0236	98.56	95.57
DRG_Tuina_1	49908500	49592802	0.024	98.44	95.21
DRG_Tuina_2	57265050	56877106	0.0239	98.47	95.32
DRG_Tuina_3	43214794	42957848	0.0235	98.59	95.66
SDH_Sham_1	52341804	51731738	0.0261	97.5	93.17
SDH_Sham_2	51960712	51356472	0.026	97.53	93.26
SDH_Sham_3	50816528	50241342	0.0262	97.48	93.11
SDH_Model_1	50957220	50388816	0.0259	97.6	93.36
SDH_Model_2	53375656	52715726	0.0261	97.5	93.19
SDH_Model_3	53827014	53217702	0.026	97.57	93.3
SDH_Tuina_1	45437160	44443632	0.0282	96.68	91.41
SDH_Tuina_2	61969304	61269358	0.026	97.57	93.32
SDH_Tuina_3	59304792	58595522	0.0263	97.44	93.03

(1) Sample: sample name, 18 Cdna libraries are sham group, model group, tuina group. (2) Raw reads and clean reads: the number of original sequence data and the data after filtering. (3) Error rate: average error rate of the sequencing base, generally below 0.1%. (4) Q20 and Q30: percentage of bases with sequencing quality above 99 and 99.9% of total bases, generally above 85 and 80%.

#### Changes of differentially expressed genes after tuina intervention

**FIGURE 3:**
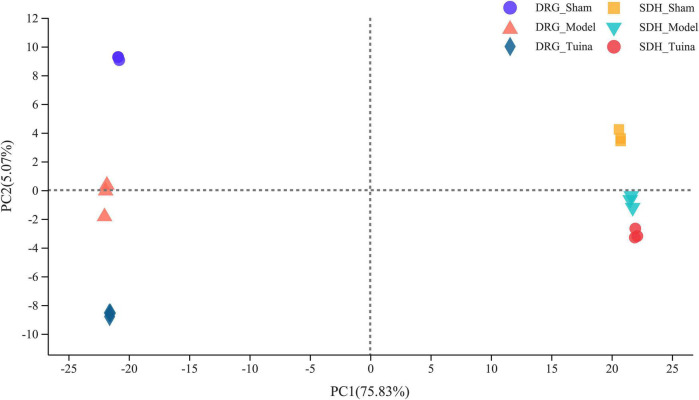
PCA analysis of DRG and SDH samples. Sham group (*n* = 3); model group (*n* = 3); tuina group (*n* = 3). PC1 = 75.83%, PC2 = 5.07%, a higher percentage means that this principal component is more capable of differentiating the samples. The distance between graphs represents how far the results of samples showed, and the farther distance indicates the lower similarity between samples.

**FIGURE 4:**
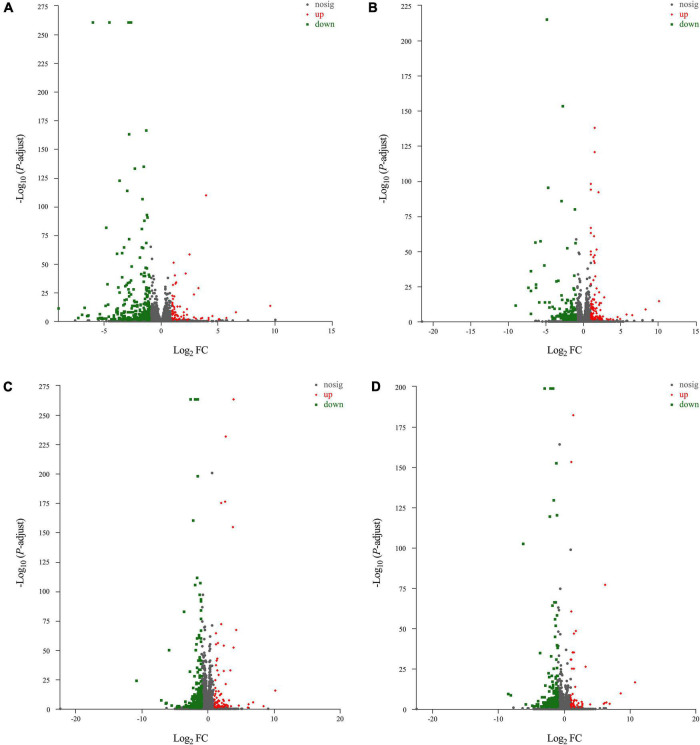
Volcano map results, the abscissa is the Log_2_ FC and the ordinate is the -LOG_10_, *P*-adjust <0.05. Results of DRG sequencing, **(A)** model *vs.* sham; **(B)** model *vs.* tuina; Results of SDH sequencing, **(C)** model *vs.* sham; **(D)** model *vs.* tuina. Gray are genes with no differential expression, red are genes with up-regulated expression, and green are genes with down-regulated expression.

**FIGURE 5:**
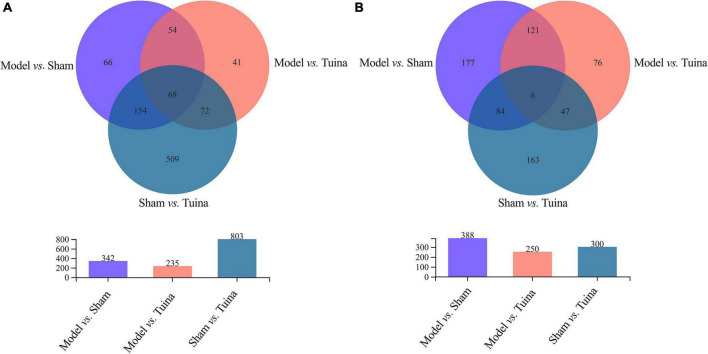
Results of the Venn analysis, the abscissa is the group, and the ordinate is the number of DEGs. **(A)** DRG sequencing; **(B)** SDH sequencing. The overlapping regions are identically expressed genes, and genes in the overlapping regions of model *vs.* sham and model *vs.* tuina are associated with the therapeutic effects of tuina.

The results of principal component analysis (PCA) showed good intragroup aggregation in each group, indicating a high degree of similarity between the samples within the group. It also showed that there was good intergroup dispersion, which indicated significant differences between all groups. The results confirmed that the model was established successfully and tuina treatment was effective (Figure 3). Changes of DEGs in DRG and SDH on the surgical side were detected by RNA sequencing. A total of 964 DEGs were detected in the DRG of the three groups. There were 65 up-regulated DEGs and 277 down-regulated DEGs in the model *vs.* sham (Figure 4A), and 106 up-regulated DEGs and 129 down-regulated DEGs in the model *vs.* tuina (Figure 4B). Venn analysis showed a total of 122 DEGs between the model *vs.* sham and the model *vs.* tuina (Figure 5A). A total of 65 DEGs showed opposite expression trends between the model group and the tuina group. A total of 674 DEGs were detected in the SDH of the three groups. There were 80 up-regulated DEGs and 308 down-regulated DEGs in the model *vs.* sham (Figure 4C), and 52 up-regulated DEGs and 198 down-regulated DEGs in the model *vs.* tuina (Figure 4D). Venn analysis showed a total of 127 DEG between the model *vs.* sham and the model *vs.* tuina (Figure 5B). 123 DEGs showed opposite expression trends between the model and tuina group.

#### Pathways analysis of differentially expressed genes regulated by tuina in dorsal root ganglia and spinal dorsal horn

**FIGURE 6:**
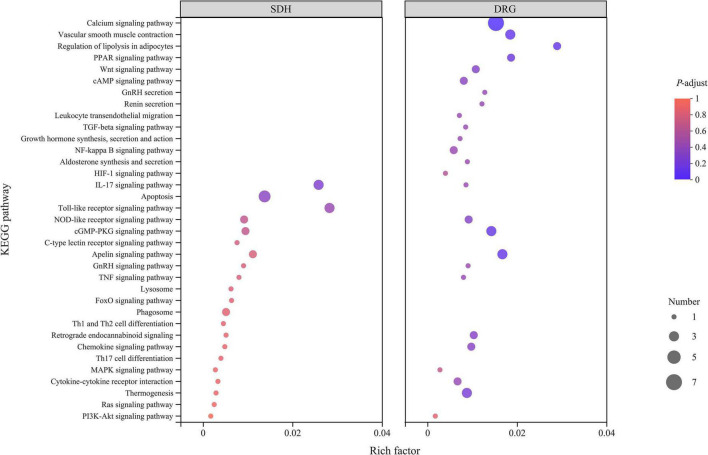
KEGG analysis results of DRG samples. SDH on the left and DRG on the right, the abscissa is the KEGG pathway, and the ordinate is the rich factor. The higher the rich factor, the greater the degree of enrichment, and the size of the dots indicates the number of genes in the pathway, while the color of the dots corresponds to the different *P*-adjust ranges.

To understand the function and role of DEGs in detail, we performed Kyoto Encyclopedia of Genes and Genomes (KEGG) analyses (Figure 6). DEGs in DRG were enriched in 75 pathways, including the calcium signaling pathway, vascular smooth muscle contraction, leukocyte transendothelial migration, regulation of lipolysis in adipocytes, gonadotropin-releasing hormone (GnRH) secretion, cGMP-protein kinase G (cGMP-PKG) signaling pathway, apelin signaling pathway, and thermogenesis pathways. DEGs in SDH were enriched in 107 pathways, including the hypoxia-inducible factor (HIF) -1 signaling pathway, interleukin-17 signaling pathway, Toll-like receptor signaling pathway, GnRH signaling pathway, mitogen-activated protein kinase (MAPK) signaling pathway, and T helper (Th) 17 cell differentiation pathways.

## Discussion

Tuina analgesia is effective and rapid, with practically no side effects (Wang et al., 2020; Rivaz et al., 2021; Liu et al., 2022). The goal of this study was to investigate the potential mechanisms in the induction of immediate analgesia by tuina. We assessed the immediate analgesic effect of tuina treatment by evaluating the changes in MWT and TWL before and after the treatment and screened DEGs using RNA-Seq. KEGG analysis was used to further explore the mechanisms.

Minor CCI model was used for simulating clinical NP. The minor CCI model causes nerve edema through ligation, producing chronic constriction and compression resulting in degeneration and necrosis of some nerve fibers which manifests as neuropathic pain with inflammatory pain after modeling, forming stable chronic pain in 3–5 days (Grace et al., 2011; MacDonald et al., 2021). In our preliminary study, consistent with the literature, we found that the model was stable at day 7 after modeling, so we chose that time point to give tuina intervention. The “Three-Manipulation and Three-Acupoint” is a combination of manipulations and acupoints that we have studied and proven to be effective (Li et al., 2019; Lv et al., 2020a). We found that after 20-time intervention, the MWT and TWL of rats with sciatic nerve injury were significantly improved, and demonstrated significant cumulative analgesic effects of the “Three-Manipulation and Three-Acupoint”.

In this experiment, we found that rats in the model and tuina groups showed significant allodynia, thermal hyperalgesia, and limp positions after modeling. The electron microscopy results of DRG and SDH in the model group also confirmed the difference in ultrastructure from the sham group. This suggests that the model replication was successful and consistent with the literature (Grace et al., 2010). While in previous experiments, we tested the therapeutic effect after a 20-time tuina intervention, the present study focuses on the immediate analgesic effect of tuina (Lv et al., 2020b). Behavioral tests were performed immediately after 1-time tuina treatment. Compared to the model group, the results of MWT and TWL in the tuina group were statistically significant. This result showed that 1-time tuina treatment can effectively alleviate hyperalgesia. Compared with mechanical hyperalgesia, the improvement in thermal hyperalgesia was more noticeable. The electron microscopic results of DRG and SDH revealed no difference in ultrastructure between the model and tuina groups, implying that short-term tuina intervention did not restore the morphology.

The calcium signaling pathway of DRG is a key factor in the immediate analgesic effect of tuina. Many of the pathways enriched in this study were associated with cascade activation of the calcium signaling pathway, which is critical to initiate and maintain peripheral sensitization of NP (Cui et al., 2021). Peripheral injurious stimuli increase intracellular Ca2^+^ through calcium signaling pathways, *N*-methyl-D-aspartic acid receptors, and other metabolic-like receptors in response to each other, and it can further activate calcium/calmodulin-dependent protein kinase II, and protein kinase A, which enhances postsynaptic excitability and triggers NP, resulting in peripheral and central sensitization (Yang et al., 2004; Latremoliere and Woolf, 2009; Incontro et al., 2018; Liu et al., 2018). The study proved that the mechanical stimulation administered to the body by tuina could initiate the skeletal muscle calcium signaling pathway, regulate the uptake and release of Ca2^+^, and adjust the intracellular Ca2^+^ concentration, thus repairing the damaged cells (Lin et al., 2013). The 1-time tuina intervention was able to modulate the calcium signaling pathway, which was beneficial in reducing nerve damage, local inflammatory microenvironment-induced nociceptive hypersensitivity, and reducing the hyperexcitability of injury-sensing neurons (Luo et al., 2008; Chen et al., 2009).

Thermogenesis/regulation of lipolysis in adipocytes in DRG and SDH is associated with the immediate analgesic mechanism of tuina. The results of behavioral tests indicated that tuina was more effective for thermal nociceptive sensitization. Tuina is considered to have a warming effect in Chinese medical theory. In the KEGG enriched pathways, we have found that the expression of thermogenesis/regulation of lipolysis in adipocytes, and uncoupling of protein-1 mediates the dissipation of energetic chemicals then induces adaptive thermogenesis through brown fat tissue. The binding to beta-adrenergic receptors increases lipolysis and regulates local energy metabolism of nerve injury at the genetic level, thereby increasing TWL (Kissig et al., 2016; Mills et al., 2018). The immediate mechanism of tuina analgesia will continue to be validated in future research. Furthermore, the KEGG enrichment results revealed that the 1-time tuina intervention was able to activate the apelin signaling pathway. Apelin is expressed in fat tissue and skeletal muscle. It can induce skeletal muscle cell proliferation and inhibit skeletal muscle atrophy, suggesting that tuina exerts immediate analgesic effects while preventing possible motor dysfunction of NP in the long term (Vinel et al., 2018).

The main limitation of this study is the lack of verification for the target genes and pathways to further confirm that the results were reliable. We will further verify the calcium signaling pathway and differential genes, focus on the gene expression changes of DRG after different times of tuina (1, 2, 3 times) in the future, and explore gene expression patterns through different therapeutic styles combined with multiple omics approaches to clarify the unique mechanism of tuina analgesia.

## Materials and methods

### Animals and ethical approval

Eighteen male Sprague-Dawley (SD) rats of 8-week-old, weighing 200 ± 10 g, were obtained from SPF Biotechnology Co., LTD. (Beijing, China), and the certificate number is SCXK (JING) 2019-0010. Rats can drink and feed freely and are cultured in an environment with the appropriate temperature (25 ± 0.5°C), humidity (45 ± 5%), and light cycle (12/12 h). All animal experimental procedures followed the local principles of the Animal Ethics Committee of the Beijing University of Chinese Medicine (BUCM-4-2020111103-4087). We strive to reduce the number of animals used and ensure their welfare in the design of our experiments.

### Modeling methods and study design

Pathological modeling started after 7 days of acclimatization. The method of modeling the minor CCI was as described in previous studies (Bennett and Xie, 1988; Grace et al., 2010). Briefly, rats were anesthetized with pentobarbital (1%, Sigma-Aldrich LLC., Germany). The sciatic nerve of the right side was exposed in front of the nerve branch by blunt dissection. A chromic intestinal suture (4-0, Shandong Boda Medical Products Co., Ltd., Shandong, China) was loosely tied around the nerve and did not interrupt the blood circulation of the epineural vasculature. The sciatic nerve was exposed for 3 min without any ligation in the sham group. The skin was then sewn together with two sutures. Seven days of acclimatization after operation, the 18 rats, which had similar levels of pain sensation, were randomly divided into the sham group (*n* = 6), model group (*n* = 6), and tuina group (*n* = 6).

### Intervention methods

The intervention was performed after the model was stably established (7th day after modeling) according to the literature and previous studies (Ma et al., 2017; Lv et al., 2020b; Talaei et al., 2022). The tuina group received a 1-time tuina intervention. The procedure for the “Three-Manipulation and Three-Acupoint” treatment was performed as follows: Firstly, a rat was fixed on the message platform of the Tuina Manipulation Simulator (Self-developed machine, China invention patent number ZL200710187403.1). Secondly, the machine parameters were set to stimulate with a force of 4 N, 60 times per minute. Third, the stimulus rod was placed on BL 37, GB 34, and BL 57 of the surgical side, then finger pressing, plucking, and kneading manipulation were stimulated, respectively. Each acupoint and manipulation were operated for 1 min consecutively for 9 min in total (Lv et al., 2020a). The grip restraint intervention was performed in the sham and model groups.

### Behavior tests

Behavioral tests were performed three times—baseline (before modeling), pre-intervention, and post-intervention. MWT and TWL were operated on the right hind paw as in the previous description (Bennett and Xie, 1988). The measurement was repeated three times with a 10-min interval between each measurement.

MWT: Measurement started when exploratory and grooming behaviors stopped, and the rats were motionless and relaxed. An electronic Von Frey instrument (BIO-EVF5; Bioseb, USA) was used, avoiding the metal grid, and the same position on the plantar surface of the hind paws was stimulated with the disposable measurement tip. The intensity of the applied force was adapted, recording the animal’s response (e.g., curling hind paws, licking hind paws) and the maximum value automatically was recorded by the system.

TWL: Using a thermal analgesia device (PL-200; Chengdu Techman Software Co., Ltd., China), the heat intensity was set to 50%, and the latency was cut off at 30 seconds to prevent skin damage. The infrared source beneath the plantar surface of the hind paws was put in the right position. The recording of the animal’s response and the time was taken automatically by the system.

### Electron microscopy

After the last behavioral test, three rats in each group were randomly selected and deeply anesthetized with pentobarbital (1%), then fixed with 4% paraformaldehyde. The DRGs and SDHs of the fourth to sixth lumbar (L_4–6_) on the right side were removed and fixed for 2 h in 0.1 M PB with 1% osmic acid. The SDH was then subjected to gradient dehydration in ethyl alcohol and acetone, with concentrations of 30, 50, 70, 80, 95, 100, 100, 100 (acetone), 100 (acetone), for 15∼20 min each. After being embedded overnight in the resin, the spinal dorsal horn was sectioned at a thickness of approximately 70 nm. Ultrathin sections were stained for 8 min with 2% uranyl acetate followed by 2.6% lead citrate for 8 min, and dehydrated overnight at room temperature. Transmission electron microscopy (Hitachi TEM system, Japan) was used for observation.

### RNA-seq

Three rats were rapidly sacrificed using the same anesthetic method. The RNA-Seq experimental procedure consists of 5 parts: (1) RNA extraction, (2) transcriptome library preparation and sequencing, (3) read mapping of raw data, (4) differential expression analysis and (5) analysis of functional enrichment and alternative splice events identification. The DRG and SDH of L4-6 segments on the right side were removed and separated from the cauda equina (stored at –80°C). Total RNA was extracted from DRG and SDH with TRIzol reagent according to the manufacturer’s protocol (Invitrogen, CA, USA), then RNA was determined in quality and quantity. Only high-quality RNA samples were selected for the next experiment. The cDNA transcriptome library was constructed following a TruSeq RNA sample preparation kit (Illumina, CA, USA) according to the protocol of the manufacturer (Chomczynski and Sacchi, 2006). The final double-stranded cDNA samples were synthesized with a SuperScript double-stranded cDNA synthesis kit (Invitrogen, CA, USA). After end-repair, phosphorylation, ‘A’ base addition, and amplification, sequencing was performed on an HiSeq X Ten platform (Illumina, CA, USA). The raw paired-end reads were trimmed, and quality controlled using SeqPrep and Sickle. The differential expression genes (DEGs) can be identified and considered statistically significant with the *P*-adjust ≤0.05 and fold change >2.0. Finally, all the alternative splice events that occurred in the samples were identified using rMATS (Shen et al., 2014).

### Statistical analysis

Data analysis was performed with SPSS Statistics software (version 26.0). MWT and TWL results were presented as mean ± SD. One-way ANOVA was used for comparisons between groups, and the LSD multiple comparison test was used for multiple comparisons. P < 0.05 was treated as statistically significant. The KEGG enrichment analysis of DEGs was performed with KOBAS.^1^

## Conclusion

In this study, we found that 1-time tuina intervention could alleviate the hyperalgesia of minor CCI rats, and especially ease thermal hyperalgesia more effectively. However, 1-time tuina treatment did not change the ultrastructure in DRG and SDH when observed using electron microscopy technology. The immediate analgesic mechanism of tuina is mainly related to the calcium signaling pathway, thermogenesis, and regulation of lipolysis in adipocytes.

## Data availability statement

The original contributions presented in this study are publicly available. The raw data of RNA-Seq can be found on NCBI at the following link: https://www.ncbi.nlm.nih.gov/bioproject/884185 with accession number: PRJNA884185. Further inquiries can be directed to the corresponding author.

## Ethics statement

The animal study was reviewed and approved by Animal Ethics Committee of the Beijing University of Chinese Medicine.

## Author contributions

TY and ZL: study conception and design of the work. HW, YZ, YX, YJ, QG, and DL: animal experiments and data acquisition. HW and ZL: analysis, data interpretation, and drafting of the manuscript. HW, ZL, and TY: approval of the final version of the manuscript. All authors contributed to the article and approved the submitted version.

## Footnotes

1
http://kobas.cbi.pku.edu.cn/home.do
